# CRISPR/Cas12a-Based Detection Platform for Early and Rapid Diagnosis of Scrub Typhus

**DOI:** 10.3390/bios13121021

**Published:** 2023-12-08

**Authors:** Pooja Bhardwaj, Nikita Shrikant Nanaware, Sthita Pragnya Behera, Smita Kulkarni, Hirawati Deval, Rajesh Kumar, Gaurav Raj Dwivedi, Rajni Kant, Rajeev Singh

**Affiliations:** 1 ICMR-Regional Medical Research Centre Gorakhpur, BRD Medical College Campus, Gorakhpur 273013, India; poojab288@gmail.com (P.B.); sp.behera1@gmail.com (S.P.B.); hirawati.deval@icmr.gov.in (H.D.); grd.rmrcb@gov.in (G.R.D.); rajnikant.srivastava@gmail.com (R.K.); 2 ICMR-National AIDS Research Institute, Bhosari, Pune 411026, India; nikitananaware20@gmail.com (N.S.N.); skulkarni@nariindia.org (S.K.); 3 RGSC, Department of Genetics and Plant Breeding, Banaras Hindu University, Varanasi 221005, India; rajeshkumar.gpb@bhu.ac.in

**Keywords:** scrub typhus, *Orientia tsutsugamushi*, rapid diagnostic kit, Gorakhpur, acute encephalitis syndrome, 56 kDa gene, CRISPR/Cas12a, lateral flow assay

## Abstract

*Orientia tsutsugamushi* is responsible for causing scrub typhus (ST) and is the leading cause of acute encephalitis syndrome (AES) in AES patients. A rapid and sensitive method to detect scrub typhus on-site is essential for the timely deployment of control measures. In the current study, we developed a rapid, sensitive, and instrument-free lateral flow assay (LFA) detection method based on CRISPR/Cas12a technology for diagnosing ST (named LoCIST). The method is completed in three steps: first, harnessing the ability of recombinase polymerase for isothermal amplification of the target gene; second, CRISPR/Cas12a-based recognition of the target; and third, end-point detection by LFA. The detection limit of LoCIST was found to be one gene copy of ST genomic DNA per reaction, and the process was complete within an hour. In 81 clinical samples, the assay showed no cross-reactivity with other rickettsial DNA and was 100% consistent with PCR detection of ST. LoCIST demonstrated 97.6% sensitivity and 100% specificity. Overall, the LoCIST offers a novel alternative for the portable, simple, sensitive, and specific detection of ST, and it may help prevent and control AES outbreaks due to ST. In conclusion, LoCIST does not require specialized equipment and poses a potential for future applications as a point-of-care diagnostic.

## 1. Introduction

*Orientia tsutsugamushi* (OT) is an obligate intracellular parasite bacteria and the causative agent of scrub typhus (ST), which is associated with acute febrile illness (AFI) [1] and transmitted by mites through an infected chigger bite (in the larval stage). This disease, which was earlier believed to be endemic in the tsutsugamushi triangle (extending from Japan and Russia in the north to Northern Australia in the south and the Arabian Peninsula in the west), has now extended from its endemic region and poses a serious global health threat to populations in countries including, but not limited to, Africa, America, the United Arab Emirates, Korea, Japan, China, Taiwan, India, Indonesia, Thailand, Sri Lanka, and the Philippines [1,2,3,4]. ST has been established as an important etiology associated with >60% of acute encephalitis syndrome (AES) cases in the pediatric population [5,6,7]. If ST is left undiagnosed and untreated, it may also lead to severe multiorgan failure, which may result in case fatalities of up to 30% or even higher [1]. ST has long been a tropically neglected infectious disease; therefore, different facets of the disease, from diagnosis to prevention, are still unclear.

ST is difficult to diagnose clinically since the symptoms resemble those of other acute febrile infections. The serological or molecular methods available for ST detection are ELISA, IFA, ICT, Weil–Felix, PCR, loop-mediated isothermal amplification (LAMP), etc. While the Weil–Felix test lacks specificity and sensitivity, other immuno-based approaches, such as IFA and ELISA, are insufficient at revealing the infection in its early stages due to the absence of detectable antibodies one to two weeks post-infection [8]. On the other hand, PCR-based approaches are highly accepted for their proficiency in early-stage diagnosis of ST, but the genetic diversity of OT hampers their applicability [8]. Based on the variations in the 56 kDa type-specific antigen, Karp, Gilliam, and Kato are considered prototypes of *Orientia*, while the genotypes include Kuroki, Shimokoshi, Kawasaki, and Boryong, etc. [9]. Among the ST genotypes, Gilliam and Karp are reported to be the prevailing genotypes of OT that lead to AFI and AES [10,11,12,13], with sporadic reports of dual infections [14]. Due to having the highest variability and being one of the immunodominant genes among the OT genotypes, the 56 kDa gene was selected for this study [15].

ST is an important neglected tropical disease affecting mainly rural populations, with an increasing effect on urban populations [16]. PCR-based approaches require expensive specialized instruments and well-established infrastructure for laboratories, as well as skilled manpower, which remain unavailable in the peripheral rural endemic areas. Thus, the unavailability of molecular rapid diagnostic tools, which can facilitate the early detection of ST, results in a delayed diagnosis and creates a delay in treatment. Keeping in mind the endemicity of ST in rural areas and the lack of infrastructure, we urge the requirement for a rapid and efficient method of ST detection.

A denaturation-free amplification method was introduced in 2006, termed recombinase polymerase amplification (RPA). In RPA, the amplification of genetic material is carried out by strand-displacing polymerase, RecA recombinase, and single-strand DNA-binding proteins (SSBs). Since the replication process is denaturation-free, the amplification of nucleic acid can be performed at a single temperature (37–42 °C) within a short duration of time (20–30 min) [17]. The ambient temperature can be provided by simple equipment such as a dry bath or a water bath. Some clustered regularly interspaced short palindromic repeat (CRISPR) systems possess trans-cleavage activity, which activates upon target recognition and has been utilized further for genome or SNP detection [18]. These CRISPR systems can be integrated with RPA to facilitate the nucleic acid detection of a low genome copy. For instance, CRISPR-based diagnostics employed with isothermal amplification, such as DETECTR and SHERLOCK, with attomolar sensitivity and single nucleotide differentiation ability, offered a new era in molecular biosensing [19,20,21]. RPA has been demonstrated to be compatible with the Cas12a endonuclease system for specific nucleic acid detection, as both can work at a common temperature (37–42 °C) [22,23,24,25].

The CRISPR/Cas12a system utilizes CRISPR RNA (crRNA) and a 5’ TTTV (V = A/G/C) protospacer adjacent motif (PAM) for target recognition. Once the target is identified by the Cas12a:crRNA nucleoprotein complex, the triggered endonuclease activity of CRISPR/Cas12a cleaves the target, as well as any non-target ssDNA present in the surrounding area [19]. The non-target DNA can be tagged by a fluorescent reporter or an immune-labeled reporter, which, upon cleavage, may result in fluorescent signal and lateral flow strip-based detection, respectively. Hence, RPA can be coupled with CRISPR/Cas12a and a lateral flow assay to facilitate end-point detection for the establishment of a robust detection method in low-resource settings, which eliminates the requirement for specialized equipment as demonstrated earlier [24,25]. PCR has long been nucleic acid’s gold standard, but the advancements in nucleic acid detection methods have led to simpler processes that rely less on well-equipped labs and trained lab staff.

In the present study, we developed a platform named **L**ateral fl**o**w detection method based on **C**R**I**SPR/Cas12a technology for **ST** (LoCIST), where RPA is coupled with CRISPR/Cas12a for the specific detection of prevalent OT genotypes, Gilliam and Karp. The sensitivity and specificity of the LoCIST were evaluated using the genomic DNA extracted from the whole blood (WB) obtained from AES cases diagnosed positive and negative for ST by IgM ELISA and other rickettsial strains and found to be comparable to that of PCR. The limit of detection of the assay was also evaluated using the copy number of 56 kDa in comparison to qPCR.

## 2. Materials and Methods

### 2.1. Materials and Reagents

The oligonucleotide sequences (Supplementary Materials) were synthesized by IDT (Coralville, IA, USA). TwistAmp Basic RPA kit and millennia hybrid detect lateral flow strips were purchased from Twistdx (Scarborough, ME, USA), Taq polymerase, high purity LbaCas12a, NEB buffer r2.1, and de-oxy nucleoside triphosphates (dNTPs) were obtained from New England Biolabs (Ipswich, MA, USA). The cloning kit pGEM-T easy vector was purchased from Promega (Madison, WI, USA). Brilliant II SYBR Green qPCR Master mix was purchased from Agilent Technologies (Santa Clara, CA, USA). The other reagents, such as buffers and media, were prepared in our laboratory.

### 2.2. Bacterial Strains

This study involved the strains of OT, including Gilliam and Karp, of which the genomic DNA was isolated from WB of well-characterized clinical samples, and other similar clinical pathogens, including standard rickettsial strains: *R. akari* from the transitional group, *R. conori* and *R. rickettsia* from spotted fever group, and *R. typhi* from the typhus group. 

### 2.3. Clinical Sample Collection and Processing

The stored clinical samples received between January 2019 and January 2020 from the AES suspected cases admitted to the BRD medical college (BRDMC), Gorakhpur, were retrieved. The 3 mL of WB for serum and 1 mL of WB EDTA were collected before initiating the antibiotic therapy with the patient’s and/or their parent’s consent. In this study, the WB samples that tested positive (n = 18) and negative (n = 63) for ST by IgM ELISA were used for validation. The cut-off OD value of the IgM ELISA assay for ST was 0.7 for serum [5].

The extraction of DNA from the patient’s WB samples was carried out using a commercial kit following the manufacturer’s instructions (Qiagen, Hilden, Germany). The isolated DNA was stored at −20 °C until further use. 

### 2.4. Positive Control Preparation

The complete ORF of the 56 kDa gene was amplified using genomic DNA extracted from the Gilliam and Karp genotypes of OT using the primer reported earlier [26] (Figure S1). PCR amplification was carried out using a Phusion™ High-Fidelity DNA Polymerase (Thermofisher, Waltham, MA, USA). The amplified 56 kDa gene after gel purification was cloned into the pGEM-T easy vector (Promega Corp., Madison, WI, USA) following the protocol described earlier [27] (Figure S2). The cloned 56 kDa ORF was further utilized for optimization of RPA as a positive control and to assess the limit of detection. 

The concentration of the recombinant plasmid was measured using a Qubit spectrophotometer (Thermo Scientific, Waltham, MA, USA). To obtain the quantitative standard curve, the copy number of the target amplicons was calculated, and the plasmid DNA was diluted to correspond to a 1–10^7^ copies per reaction. The copy number of the intended target was calculated using the following formula:

(1) $$DNA\left(\frac{copies}{mL}\right)=\frac{\left[\left(6.023*10^{23}\right)*c*10^{-9}\right)]}{DNA\;length\left(bp\right)*660}$$

where

6.023 ∗ 10^23^. is Avogadro’s constant;

660 = Average mass of 1 basepair (bp) dsDNA;

DNA concentration (c) = OD_260_*dilution factor.

### 2.5. Isothermal Amplification with RPA

The RPA primers for the 56 kDa gene were designed in-house according to the Twistdx instruction’s manual; they were 35 nucleotides (nt) long with an amplicon length of 200–400 bp. These primers were screened for secondary structure, homo-dimer, cross-dimerization, and possible hairpin using IDT Oligo Analyzer (https://www.idtdna.com/pages/tools/oligoanalyzer, accessed on 24 February 2023) and net primer (http://www.premierbiosoft.com/NetPrimer/AnalyzePrimerServlet, accessed on 24 February 2023). The optimum primer pair was selected for 56 kDa gene, and amplification was performed using the TwistAmp Basic Kit with the same primer set as the qPCR. Concentrations of the primers in RPA were optimized ranging between 200 and 480 nM. The total RPA reaction volume was adjusted to 25 μL. Briefly, the lyophilized RPA pellet was resuspended in 29.5 μL rehydration buffer mixed with 0.48 µM of each forward and reverse primer and 1 μL of the template, followed by the addition of 14 mM magnesium acetate in the tube lid and spun down to start the reaction. The target was amplified by incubating the reaction at 39 °C for 20 min in a dry bath. The list of DNA and crRNA sequences used in this study is provided in Table S1.

The amplified products of RPA were cleaned through column purification by QIAquick gel extraction kit (Qiagen, Germany) with minor modifications (Figure S3) to confirm amplification. The RPA end products were directly mixed with 2 volumes of isopropanol followed by a column purification step as per the instruction manual. The cleaned RPA products were subjected to agarose gel electrophoresis. Subsequently, the band of interest was excised and purified, and the specificity of RPA was confirmed by sequencing. Further, the product of RPA was directly used for CRISPR/Cas12a-based endpoint detection through lateral flow assays.

### 2.6. CRISPR RNA (crRNA) Synthesis and CRISPR/Cas12a-Based Detection of ST

A pair of CRISPR RNA (crRNA) for the target was designed manually for specific detection of the 56 kDa gene sequence of OT adjacent to the 5′-TTTV protospacer adjacent motif (PAM) site. The optimized cr56kDa2 sequence was 5′-UAAUUUCUACUAAGUGUAGAUAUCUGAGUAUGAUUGUUGGCC-3′, consisting of a scaffold 5′-UAAUUUCUACUAAGUGUAGAU-3′ and spacer RNA 5′-AUCUGAGUAUGAUUGUUGGCC-3′ (Table S1).

While the RPA reaction was in progress, the CRISPR/Cas12a reaction was set up in a total volume of 30 μL containing 1X r2.1 buffer and a 1:1 volume of Cas12a:crRNA and incubated at room temperature or 25 °C for 10 min to facilitate Cas12a:crRNA complex formation. Further, 50 nmole of ssDNA reporter and 2 μL of RPA product were added to the pre-assembled Cas12a:crRNA complex and subsequently incubated at 37 °C for 20 min to ensure complete cleavage of the reporter molecule.

### 2.7. RPA-CRISPR/Cas12a Coupled with a Lateral Flow Detection

For ease of interpretation, an end-point assay with a lateral flow test strip was developed for OT detection, integrating RPA-CRISPR/Cas12a. The FAM/biotin-labeled ssDNA reporter molecule was synthesized from IDT and lateral flow strips; HybriDetect (Milenia Biotec, GmbH, Versailles, Germany) was used to capture labeled nucleic acids. For end-point detection, the RPA:Cas12a:crRNA reaction was mixed with 100 μL of running buffer provided with the strips, and the lateral flow strip was immersed in the solution. Uncleaved reporter molecules were captured at the first detection line (test line), whereas the indiscriminate ssDNA cleavage activity of CRISPR/Cas12a did not generate any signal at the first detection line but generated only a signal at the second line (control line). The result was recorded immediately (<3 min) to avoid any false interpretation, and the image was captured using a cell phone camera.

### 2.8. Establishment of Sensitivity and Specificity of LoCIST

Using cloned 56 kDa:pGEM-T plasmid isolated from recombinant colonies, the analytical sensitivity of the assay was assessed. As stated in Section 2.3, 56 kDa recombinant plasmid DNA was serially diluted from a range of concentration between 1 and 10^7^ copies µL^−1^. The test line (T-line) signal in the LFA with the lowest copy number was detected to determine the LOD. To determine the analytical specificity of the LoCIST, clinical samples obtained from AES patients were used.

### 2.9. Real-Time PCR

The standard curve for copy number quantification was performed using a 2X Brilliant SYBR green mix kit (Applied biosystem, Waltham, MA, USA) as per the manufacturer’s instructions. Serially diluted concentrations of 56 kDa recombinant plasmid DNA, 1, 10, 10^2^, 10^3^, 10^5^, and 10^7^ copies per reaction were used as a template. A negative control was included, where template DNA was replaced with nuclease-free water. All reactions were set up in triplicate. Quantitative PCR (qPCR) analysis was performed using the Biorad CFX96 Real-Time PCR system. The cycling condition includes 95 °C for (10 min) and 40 cycles of 95 °C (30 s), 60 °C (60 s), and 72 °C (30 s). Melt curve analysis was performed at the end of the PCR, starting with 55 °C for 10 s with an increment of 0.5 °C. Microsoft Excel, MS Office 10 was used to calculate the regression coefficients (R^2^). The threshold cycle values against the copy number were plotted using scatter plot and the trendline was added to calculate the R^2^ value. A R^2^ value equal to or higher than 0.99 was considered significant.

### 2.10. Bioinformatical Tools

Primers were designed using the complete 56 kDa gene sequences retrieved from NCBI GenBank. The alignment of the nucleotide sequences was performed using ClustalW in MEGA-X [28]. The consensus sequence was obtained using Jalview [29]. The designed primers were cross-verified for the intended target using primer blast before synthesis [30].

## 3. Results

### 3.1. Construction of the Recombinant Plasmid 

The recombinant plasmids of Gilliam and Karp 56 kDa–pGEM-T constructs were digested using restriction enzymes *SpeI* and *SacII* and analyzed by agarose gel electrophoresis. Two fragments of the bands at sizes of ≈3000 bp and ≈1500 bp were observed, which were in accordance with the size of the plasmid pGEM-T easy and the target gene, respectively (Figure S2). Further, the target gene in the recombinant plasmids was also confirmed by sequencing. The sequences can be found at GenBank under accession numbers MZ292564 (Karp) and MZ292589 (Gilliam).

### 3.2. Establishment of the LoCIST Platform

**Figure 1:**
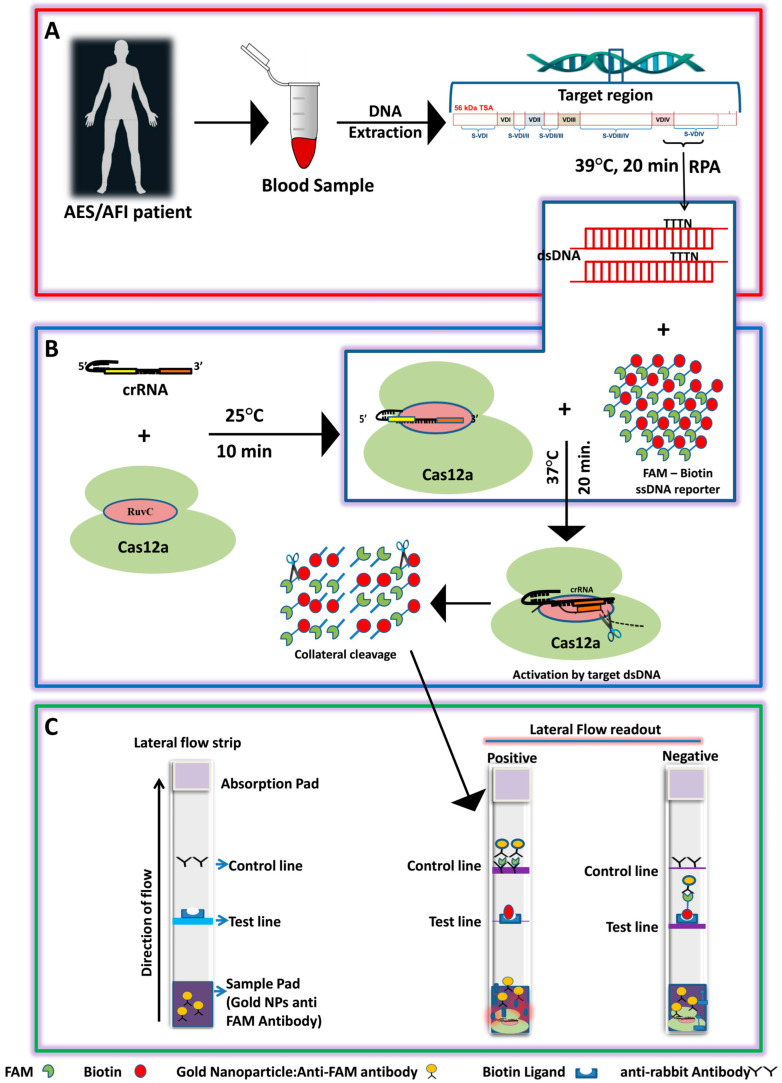
Schematics of the LoCIST platform working principle. (**A**) Extraction of DNA and the region targeted for detection of *Orientia tsutsugamushi* by RPA; (**B**) CRISPR/Cas12a-mediated cis and trans-cleavage upon target recognition. (**C**) Lateral flow assay for endpoint detection of OT.

In this study, we have developed a quick and sensitive platform termed “LoCIST”, which is a lateral flow assay based on CRISPR/Cas12a detection of the OT (Figure 1). Among the in-house-designed RPA primers, 1115F and 1419R demonstrated optimum results and were selected to carry out further experiments for the CRISPR/Cas-based detection of OT (Figure S3). 

**Figure 2:**
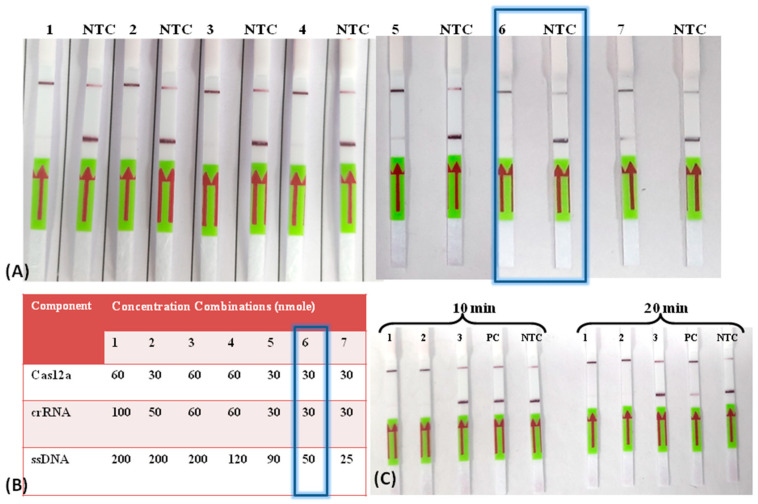
Optimization of Cas12a, cr56kDa2, and ssDNA reporter for lateral flow detection of ST. (**A**) CRISPR/Cas-LFA-based detection of ST at different concentration combinations of Cas12a, cr56kDa2, and ssDNA reporter cr56kDa2. (**B**) Table showing the different combinations of Cas12a, cr56kDa2, and ssDNA reporter concentrations used (optimal concentration highlighted with box). (**C**) Time optimization for CRISPR/Cas12a reaction at optimized concentration of Cas12a, cr56kDa2, and ssDNA. The image was captured after 10 min and 20 min of reaction completion. Each set includes 2 positive clinical samples (1–2), 1 negative control for ST (3), positive control (100 gene copies µL^−1^ of plasmid template) labeled as PC, and a no template control (NTC).

Two crRNAs (cr56Kda1 and cr56Kda2) were designed manually from the region adjacent to the PAM sequence from the sense (5′-TTTV) and anti-sense (3′-TTTV) strands, respectively, for the identified target region (Table S1). The cr56Kda2, which was located near 5′-TTTV, demonstrated a more promising result on the lateral flow assay (LFA) than the other one (Figure S4). The product of the CRISPR/Cas12a reaction before and after the completion of the CRISPR/Cas12a reaction indicated complete degradation of the target product amplified during RPA, demonstrating the ideal performance of the CRISPR/Cas12a reaction (Figure S5). Further, the concentrations of Cas12a, cr56Kda2, and ssDNA octamer Biotinylated-FAM reporters (5′-6FAM/TTATTATT/Biotin-3′) were optimized in such a way that the positive test would be evident as a clear or faint test band whereas, in the negative test, both bands would be evident. The optimized concentration for such a result was found to be 30 nmole, 30 nmole, and 50 nmole for Cas12a, cr56Kda2, and ssDNA reporter, respectively (Figure 2A,B). The incubation time for CRISPR/Cas12a was also checked, taking 100 gene copies per reaction of cloned plasmid as an input template. The results demonstrated that the time duration of 20 min was enough to detect the low copy of ST genomic material (Figure 2C).

### 3.3. Detection Limit of LoCIST

**Figure 3:**
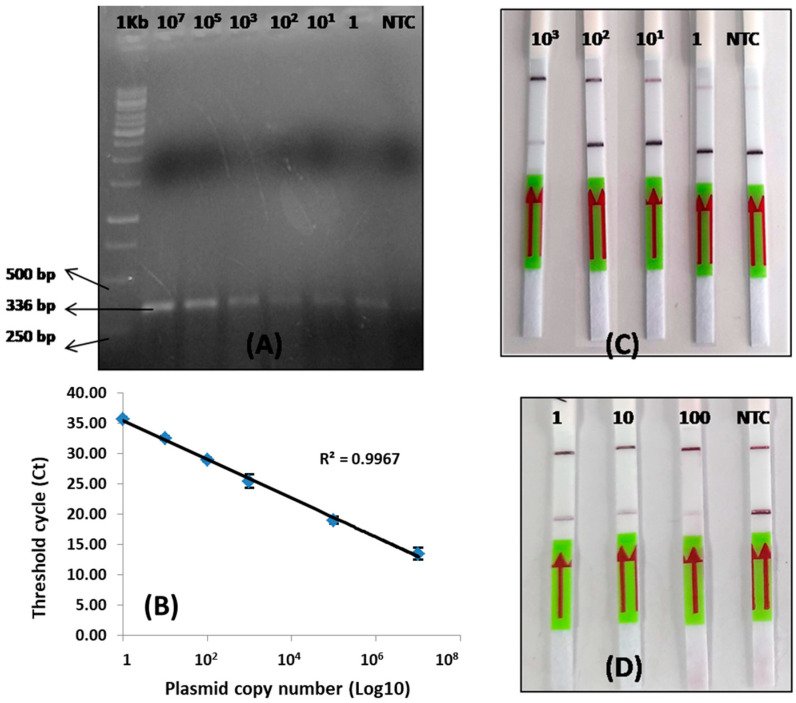
Limit of detection for LoCIST. (**A**) Gel image of the qPCR product with different 56 kDa gene copy number. (**B**) Scatter plot with trendline showing the threshold cycle (Ct) value obtained with respective gene copy number; (**C**) CRISPR/Cas12a and lateral flow detection at a concentration of 250 nmole, 62.5 nmole, and 500 nmole for Cas12a, cr56kDa2, and ssDNA reporter, respectively; (**D**) CRISPR/Cas12a and lateral flow detection at a concentration of 30 nmole, 30 nmole, and 50 nmole for Cas12a, cr56kDa2, and ssDNA reporter, respectively.

The linear standard curve from qPCR demonstrated a R^2^ value of 0.99, which is statistically significant (Figure 3A,B). This CRISPR/Cas12a-based platform was optimized at a varying range of concentrations of Cas12a, cr56kDa2, and ssDNA. Two alternative combinations of Cas12a, cr56kDa2, and ssDNA concentrations: (1) 50 nmole, 62.5 nmole, and 500 nmole, as previously described [31], and (2) 30 nmole, 30 nmole, and 50 nmole, were shown to provide optimal results on the lateral flow strip, but in two different ways. Combination 1 produced a test band on the negative sample and both a control and a test band on the positive samples (Figure 3C). Whereas combination 2 resulted in the development of both the control and test lines on the negative sample and only a sharp control band and a low (low target gene copy) or no T-line (high target gene copy) on the positive samples (Figure 3D). 

Combination 1 was tested for gene copy numbers ranging between 1 and 1000 copies per reaction, and combination 2 was tested for gene copies per reaction ranging between 1 and 100. Both combinations demonstrated the detection of one copy per reaction. Hence, the limit of detection demonstrated by LoCIST is up to one gene copy (Figure 3C,D). Combination 1 employs a high concentration of Bio/FAM-labeled ssDNA reporters to block all the anti-FAM antibodies at the T-line, leaving the control line (C-line) clear. Only upon CRISPR/Cas12a activation does the FAM analyte reach the C-line, and hence both C- and T-lines developed and indicated positive results, leaving some reporters uncleaved (Figure 3C). However, when the target is present in ample quantity, all reporters become cleaved, leaving the T-line clear. Upon subsequent repetition the similar results were not obtained with the combination 1 concentration. Hence, the concentration was further optimized and concentrations of Cas12a, cr56kDa2, and ssDNA reporter in combination 2 produced the optimum and reproducible results with three replicates. Therefore, further optimization for LoCIST development was carried out using combination 2 concentrations, where the presence of C-line was indicative of a negative result and the absence of C-line was an indication of a positive result.

### 3.4. Clinical Specimens

The samples were collected on the same day of patient admission and stored at −20 °C until further use. A total of 81 clinical samples tested positive and negative by IgM ELISA and PCR for ST were used for validating the developed LoCIST platform. Among the samples that tested positive by PCR (n = 43), the female-to-male ratio was 1:1.5. The majority of the population comprised the pediatric age group 1–10 years (67.4%). The average time interval between the date of fever onset and the date of sample collection was found to be 8.75 days for ST ELISA IgM-positive samples and 5.83 days for ST ELISA IgM-negative and PCR-positive samples (Table 1).

### 3.5. Performance of the LoCIST with Clinical Samples 

**Figure 4:**
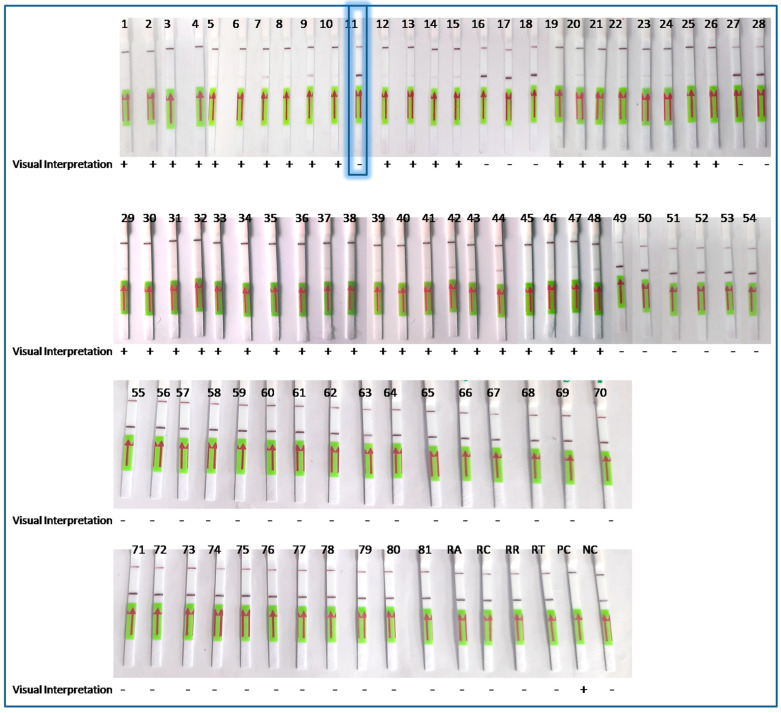
Sensitivity and specificity check of LoCIST platform with clinical specimens where positive (+) (n = 43); negative (−) (n = 38); RA = *R. akari*; RC = *R. conori*; RR = *R. rickettssi*; RT = *R. typhi*; PC = positive control (pGEMT:56kDa plasmid); NTC = no template control. In the figure, the highlighted box represents the sample (number 11) detected as false-negative by the LoCIST platform.

The LoCIST platform, along with IgM ELISA, was compared with conventional PCR targeting the same 56 kDa gene. With 81 AES clinical specimens, which included IgM ELISA-positive or PCR-positive for ST (n = 43) and IgM ELISA- and PCR-negative for ST (n = 38), the sensitivity and specificity of the LoCIST platform were established. The LoCIST platform demonstrated 97.67% sensitivity and 100% specificity when compared to PCR (Figure 4). 

### 3.6. Unambiguous Detection of OT by cr56kDa2

In silico analysis of cr56kDa2 revealed that the region is conserved among *Orientia* genotypes, including the prevalent genotypes, Gilliam and Karp, in India. As predicted, cr56kDa2 could precisely target the prevalent *Orientia* species. To verify its specificity on target, we tested our diagnostic platform with the strains of *Orientia*, Gilliam and Karp, along with the other rickettsial species, including *R. akari*, *R. conori*, *R. rickettsia*, and *R. typhi*. The results demonstrated that LoCIST could precisely detect OT strains, Gilliam and Karp, without showing any cross-reactivity with other rickettsial strains. Overall, cr56kDa2 showed 100% specificity and could be used for the detection of OT strains from various geographical regions of India.

## 4. Discussion

The OT is associated with ST disease, which accounts for >60% of the total AES cases admitted to the hospital among the reported etiologies [5]. Being the common cause of fever in AES and AFI patients, delayed diagnosis of ST may contribute to multiple organ dysfunction and up to a 25% increased mortality rate, specifically in the pediatric population [32]. Hence, a rapid diagnostic test with at least 70% sensitivity is required [33]. The sensitivity of molecular tests, including PCR and real-time PCR, over conventional immunoassay has been well established; nevertheless, they are only available in centralized laboratories. The existing gold standard for molecular detection of ST includes PCR, nested PCR, or real-time PCR. The limitation associated with the current gold standard is that it requires high-quality genomic material, which is a tedious process. Moreover, the complete process, from sample processing to its detection, requires sophisticated instruments, such as a high-speed centrifuge, a thermal cycler, good infrastructure, and skilled manpower [34]. Whereas isothermal amplification via RPA has provided a new horizon to rapid molecular tests, eliminating the requirements of sophisticated instruments, and it can be conducted with crude DNA; hence, it does not require a high level of sample processing for obtaining genomic material [35,36]. Therefore, it serves as an ideal candidate for the point-of-care aspect.

Based on isothermal detection, i.e., RPA [33,37,38] or LAMP [39,40,41] integrated with either a visual, fluorescent, or lateral flow test of ST, were developed earlier (Table 2). The sensitivity and specificity of these diagnostic approaches made using isothermal amplification alone, either with LAMP or RPA, demonstrated sensitivity ranging from 20 to 96.1% and a specificity ranging from 99.16 to 100% [39,40,41]. These developed isothermal diagnostic assays targeted either GroES [41], 56 kDa [38], or 47 kDa gene [37,42]. Since the PCR based on 56 kDa demonstrated high specificity (94.9–100%) over 47 kDa and the GroEL-based PCR assay and being the major immunodominant protein located on the outer membrane of the bacteria [43,44], 56 kDa gene was targeted in this study rather than other genes. The CRISPR/Cas-specific cleavage activity can be engineered and repurposed for the identification of a specific target [21]. Using deactivated CRISPR/Cas9, a dCas9-mediated biosensor has been developed by Koo et al. for severe fever with thrombocytopenia syndrome (SFTS) and ST detection [45]. But, firstly, the suggested platform has not defined the specific OT strain detected. Secondly, the platform result is based upon the changes in refractive index (RI), which further requires an instrument to capture the change in RI, while the visual detection can be readily interpreted by any personnel.

The cis- and trans-cleavage properties of CRISPR/Cas12a have been extensively harnessed for the development of molecular detection-based diagnostic platforms [19,21,47,48]. Utilizing this astonishing property of the Cas12a system, we have designed a LoCIST platform integrated with RPA and LFA. The RPA was able to generate a billion copies of the target gene in record time at an isothermal temperature (37–40 °C) without the use of sophisticated instruments [17]. LbCas12a isolated from the *Lachnospiraceae bacterium* has been shown to act best at the same temperature as RPA and has been widely accepted for the development of detection platforms over other Cas12a endonucleases such as AsCas12a from *Acidaminococcus* sp. and CeCas12a from *Coprococcus eutactus* [49,50]. Therefore, we have employed an engineered form of LbCas12a for the development of our detection platform. Cas12a, upon target recognition with the help of engineered crRNA, activates its cis-cleavage (target-specific). followed by trans-cleavage activation (non-specific) [19]. In LoCIST, coupled RPA and CRISPR/Cas12a provided the advantage of triple specificity: firstly, target recognition by a specific primer; secondly, with crRNA-directed CRISPR/Cas12a target recognition activating target cleavage; and lastly, the reporter cleavage (non-target), which will occur only in the presence of a specific target. Hence, the target is recognized twice in the overall process of target detection, providing the utmost sensitivity and specificity. The trans-cleavage (FAM/biotin-labeled ssDNA reporters) activity of CRISPR/Cas12a results in enhanced signal output in LFA. Overall, LoCIST demonstrated its superiority over the earlier reported platform in terms of sensitivity, specificity, and limit of detection (Table 2). The novelty of this approach lies in the coupling of RPA-CRISPR/Cas12a-LFA, and hence, this is the first report to detect OT utilizing the Cas12a endonuclease system. LoCIST provided an easy-to-use platform for OT detection. 

crRNA, a short stretch of nucleotides (18–20 bases) complementary to the target and present near the PAM motif (5′-TTTV), only activates the Cas12a endonuclease activity, cleaving arbitrarily the ssDNA present in the vicinity [19]. However, PAM-free target detection by LbCas12a has also been reported [36,51]. To evaluate the stringency of the PAM sequence, we designed two crRNAs, one present near 3′-TTTV (cr56kDa1) and the other near 5′-TTTV (cr56kDa2). As described earlier, among the two, only cr56kDa2 near 5′-TTTV was able to activate the Cas12a endonuclease, demonstrating the robustness of Cas12a detection. These results support the earlier reports [25,47,49]. In continuation, we have modified the method of detection such that the presence of both C- and T-lines indicates a negative result, whereas the absence of a T-line indicates a positive result. Thus, when target detection is facilitated by engineered cr56kDa2, the activation of Cas12a endonuclease occurs, which results in the cleavage of complete reporters at optimized concentrations of Cas12a (30 nmole), cr56kDa2 (30 nmole), and ssDNA reporters (50 nmole). Upon cleavage, all cleaved reporters could travel to the C-line, leaving the T-line clear, hence indicating a positive test. A faint or low T-line indicates a positive sample with a low concentration of the genome target. Further, the lateral flow strip procured for the assay was meant for the detection of a dual-label incorporated target, giving a signal at the T-line, whereas the C-line always develops as the labels are present individually [52]. Due to the cleavage of the dual-labeled ssDNA reporters by CRISPR/Cas12a, the C-line develops, whereas the T-line diminishes. Consequently, the results were reproducible regardless of the number of repetitions of the assay.

We found that the average time interval between the date of fever onset and the date of sample collection was 8.75 days for ST ELISA IgM-positive samples and 5.83 days for ST ELISA IgM-negative and PCR-positive samples. Our result is supported by earlier studies demonstrating the presence of insufficient detectable antibodies up to 7–14 days post-infection [8]. We assume that the antibody titer was too low to reveal the infection by ELISA IgM, as the interval window between fever onset and sample collection was too short. On the contrary, the shorter interval between fever onset to sample collection was too favorable for molecular detection, i.e., nucleic acid-based detection of the target before the initiation of therapeutic interventions [26,53]. The LoCIST could precisely detect the presence of OT in clinical samples that were detected as negative by ELISA; hence, the platform demonstrated its ability to detect the target at the earliest. We have established this rapid diagnostic tool for OT detection for up to one 56 kDa gene copy per reaction. Further, this platform readily detects the OT genotypes prevalent in India, such as Gilliam and Karp, thus facilitating the detection of these neglected tropical diseases in AES cases in remote regions. Additionally, LoCIST demonstrated its sensitivity and specificity with clinical samples, including both positives and negatives for ST. Furthermore, LoCIST can easily differentiate among the closely related genera of OT, i.e., rickettsia. Further, to deploy this technology for on-site diagnosis, simplified and instrument-free sample processing (DNA extraction) is required, where the current study is lagging. Studies have demonstrated the utilization of low resources for sample preparation, such as heat lysis, chemical lysis, magnetic nanoparticle-based, and FTA card-based [54,55,56]. Once the DNA extraction process becomes simplified, the LoCIST employing the RPA-CRISPR/Cas12a-LFA assay would enable the bedside detection of OT in remote areas.

The rapid diagnostic tests available for ST diagnosis are serology-based, and the rise of antibody titers to detectable levels takes time (>7 days). By the time the diagnosis is confirmed, the patient may have already entered the phase of severity. Nucleic acid detection is precise, and it can aid in the early detection of OT and prevent the progression of disease. Here, we provided a precise solution to the delayed diagnosis and treatment of ST in low-resource settings, which is the requirement for real-world challenges. The coupling of CRISPR/Cas12a-based molecular detection to RPA and LFA provided advancements in the molecular diagnostic approach reported earlier. LoCIST holds a potential rapid diagnostic tool as a valid molecular approach, but it is not restricted to peripheral rural and semi-urban settings. It may facilitate the early diagnosis of ST because of its simplicity and results comparable to PCR. Furthermore, LoCIST can enable clinical practitioners to initiate timely therapeutic intervention and can also be deployed in rudimentary mobile laboratories, some basic medical units, such as those in primary health care clinics, surveillance, and epidemiological studies.

## Figures and Tables

**Table 1:** Demographic details of the available AES case data detected positive by PCR.

Feature	Number (n)	Percentage (%)	Total Data Availability (n)
Sex			
Male	26	60.46	43
Female	16	39.53	
Age group			
1.1–5	16	37.20	43
5.1–10	13	30.236	
10.1–15	7	16.27	
15.1–20	2	4.65	
20.1–30	4	11.62	
Clinical symptoms			
Fever Grade			
High	20	60.6	33
Low	7	21.21	
Vomiting	18	54.54	
Loose motion	5	15.15	
Abdominal pain	7	21.21	
Headache	9	27.27	
Retrorbital	1	3.03	
Cough	2	6.06	
Altered Sensorium	15	45.45	
Seizure	1	3.03	
Body tightening	19	57.57	
Frothing	12	36.36	
Up rolling of eyeball	16	48.48	
Abnormal behavior	1	3.03	
Time interval between symptoms onset and DOA			
ELISA positive	8.75	16	40
ELISA negative	5.8	24	

DOA = Date of admission.

**Table 2:** Sensitivity and specificity of the available scrub typhus isothermal detection platform.

S. No.	Platform	Isothermal Amplification	CRISPR/Cas System	End Point Detection	Gene Target	Sensitivity	Specificity (%)	LOD	Reference
1	LAMP assay	LAMP	-	Agarose gel electrophoresis/real-time	groEL	-	-	3 gene copies	[39]
2	47-RPA-nfo and 47-RPA-Exo	RPA	-	Lateral flow/fluorescence	47 kDa	80%	100	10–60 gene copies	[37]
3	dCas9 based SMR	RPA	dCas9	Resonance-based micro rings	56 kDa	1 copy	100	0.54 aM	[45]
4	RPA-LF	RPA	-	Lateral flow assay	56 kDa	100%	90	10 gene copies	[38]
5	Real time-RPA	RPA	-	Real-time fluorescence	47 kDa	80%	100	100 gene copies	[33]
6	RPA	RPA	-	Fluorescence	56 kDa	-	-	-	[46]
7	SFTSV/OT/IC RT-LAMP	LAMP	-	Real-time fluorescence	-	91.6%	100	2^5^	[41]
8	LoCIST	RPA	Cas12a	Lateral flow assay	56 kDa	97.6%	100	1 gene copy	This study

## Data Availability

Data is contained within the article and supplementary material.

## Supplementary Materials

The following supporting information can be downloaded at https://www.mdpi.com/article/10.3390/bios13121021/s1, Figure S1: Gel image showing complete OT-56kDa ORF amplified gene from Gilliam and Karp strain of *Orientia tsutsugamushi*. Figure S2: Gel image showing (A) restriction digestion of the recombinant plasmid with *SpeI* and *SacII*. (B) Plasmid isolated from the transformed colony. (C) PCR using plasmid as template. Figure S3: Gel image showing (A) RPA with different in-house designed RPA primers with OT strain Gilliam and Karp. (B) RPA PCR-purified products respective of gel image A. Figure S4: CRISPR/Cas12a-based detection of ST. The 56 kDa recombinant plasmid with 1000 copies per reaction was used for the optimization of the detection platform. Figure S5: Gel image showing the CRISPR/Cas12a reaction before and after the CRISPR/Cas12a reaction. Table S1: List of primers used in this study. Table S2: Target amplicon sequence with forward and reverse primer highlighted. Video S1: CRISPR/Cas12a-based Lateral flow detection. In the video from left to right, one–two clinically positive samples of scrub typhus, three positive controls of scrub typhus, four negative controls, and five no template control.
