# Supplementary material for: CRISPR/Cas12a-Based Detection Platform for Early and Rapid Diagnosis of Scrub Typhus
Source: Biosensors (Basel). 2023 Dec 8;13(12):1021. doi: 10.3390/bios13121021 (PMC10742217; doi:10.3390/bios13121021)
Supplement: Supplementary file 1 [file biosensors-13-01021-s001.zip › Supplementary Data.pdf]

Supplementary Information

CRISPR/Cas12a- Based Detection Platform for Early and Rapid Diagnosis of Scrub Typhus

Pooja Bhardwaj[1], Nikita Nanaware[2], Sthita Pragnya Behera[1], Smita Kulkarni[2], Hirawati Deval[1], Rajesh Kumar[3], Gaurav Raj Dwivedi[1], Rajni Kant[1], Rajeev Singh[1]*

[1]ICMR-Regional Medical Research Centre Gorakhpur, BRD medical college campus, Gorakhpur, 273013, India

[2]ICMR-National AIDS Research Institute, Bhosari, Pune, 411026, India

[3]RGSC, Banaras Hindu University, Department of Genetics and Plant Breeding, Varanasi-221005, U.P. India

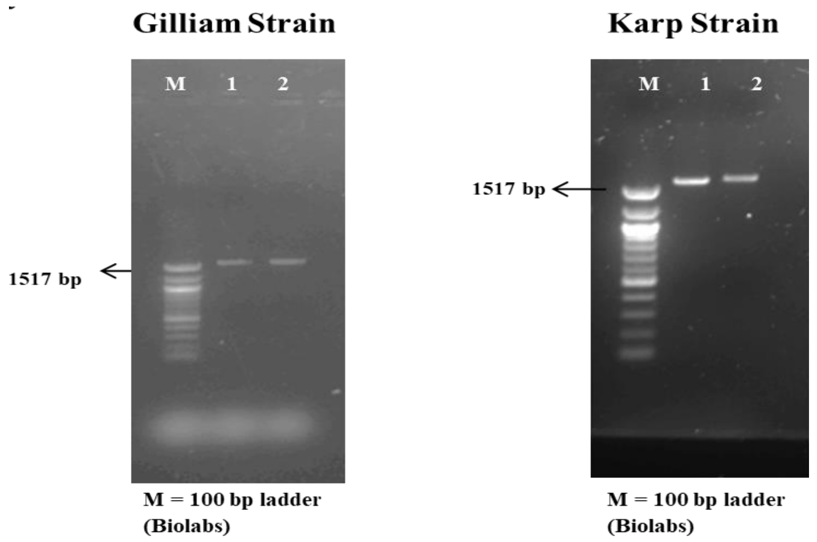

Figure S1: Gel image showing complete OT-56kDa ORF amplified gene from Gilliam and Karp strain of *Orientia tsutsugamushi*.

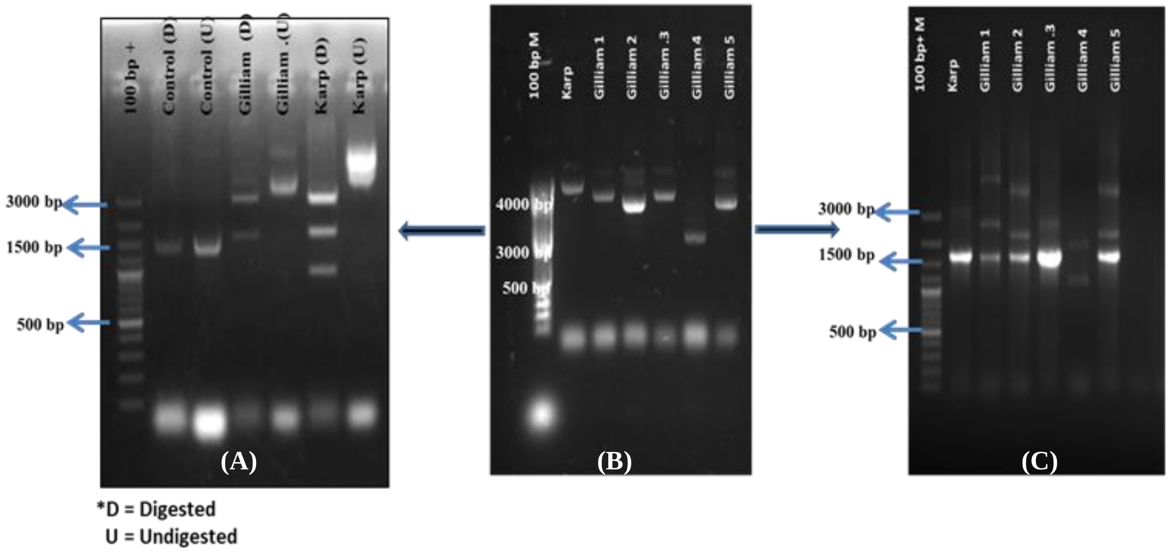

Figure S2: Gel image showing (A) Restriction digestion of the recombinant plasmid with *SpeI* and *SacII*. (B) Plasmid isolated from the transformed colony. (C) PCR using plasmid as template.

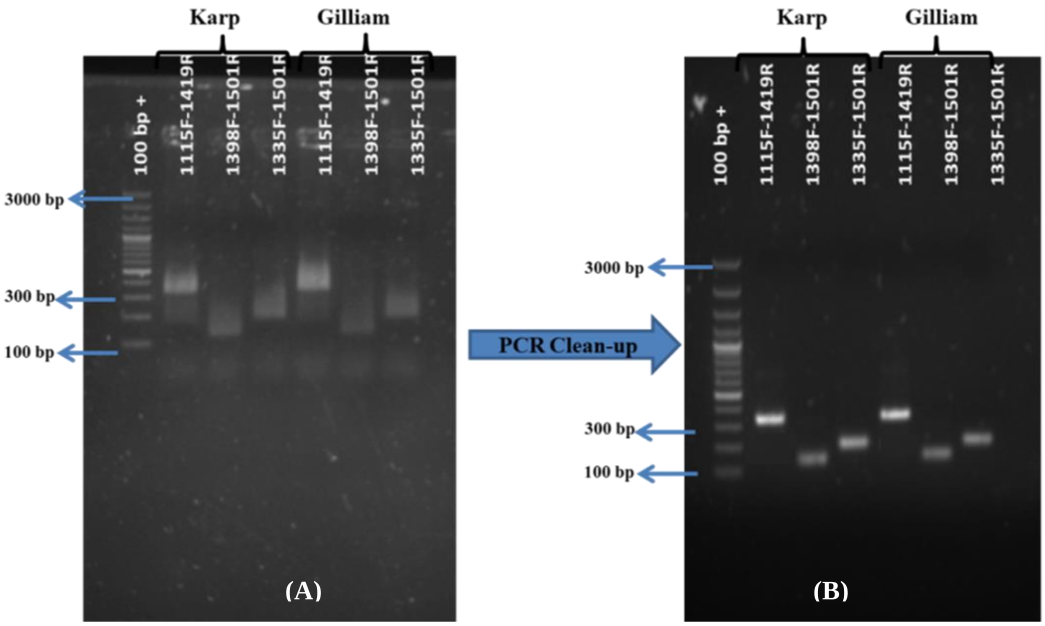

Figure S3: Gel image showing (A) RPA with different in-house designed RPA primers with OT strain Gilliam and Karp. (B) RPA PCR-purified products respective of gel image A.

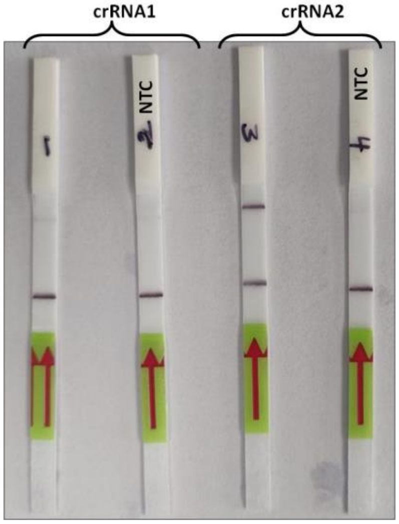

Figure S4: CRISPR/Cas12a-based detection of ST. The 56 kDa recombinant plasmid with a 1000 copies per reaction was used for the optimization of the detection platform.

Component	1	2	3	4
**Target**	+	-	+	-
**Cas12a**	-	-	+	+
**crRNA2**	-	-	+	+
**Reporter**	-	-	-	-

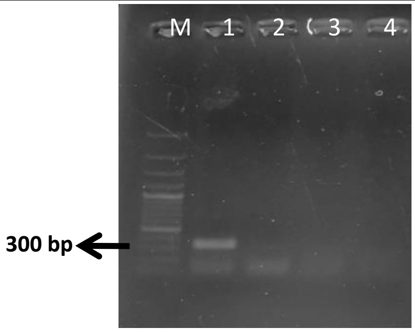

Figure S5: Gel image showing the CRISPR/Cas12a reaction before and after the CRISPR/Cas12a reaction.

Table S1: List of primers used in this study

S. No.	Primer Name	Purpose	Sequence 5'>3'	Reference
1	TSA56F1	56 kDa gene Complete ORF	AAGAATGAAAAAAATTATGTTAATTGC	(Bhardwaj et al., 2023)
2	TSA56R1		AAAACTAGAAGTTATAGCGYACAC	
3	cr56kDa1 (1475-1495)	56 kDa CRISPR/Cas-LFA	UAAUUUCUACUAAGUGUAGAUCAUAAGUAUGAGCUAACCCU	This study
4	cr56kDa2 (1381-1401)		UAAUUUCUACUAAGUGUAGAUAUCUGAGUAUGAUUGUUGGCC	This study
5	ssDNA Cas12a		5'-6FAM/TTATTATT/Biotin-3'	(Sun et al., 2021)
6	OTS1115F	56 kDa RPA	GATCTTGTTAARTTGCAGCGTCATGCAGGA	This study
7	RPA1419R		TGCTACACCAAGTGCYCCTGATGCAACCATG	This study
8	OTS1398F		GGATATTAAAGSGCATACAGGCATGGTTGC	This study
9	OTS1335F		ATTCTCAATATATGCTGGTSYTGGTGCAGG	This study
10	OTS1501R		TGARTACTTCTCTTCTATTTTACTGAATGA	This study
11	OTS1268F		CAGAGTTTGATCTGAGTATGATTGTCGGC	This study
12	OTS1448R		GCTACACCAAGTGCTACTGATGCTACTATG	This study

Table S2: Target amplicon sequence with forward and reverse primer highlighted

Complete Gene ORF	AT**GAAAAAAATTATGTTAATTGC**TAGTGCAATGTCTGCATTGTCATTGCCGTTTTCAGCTAGTGCAATAG<br>AATTGGGTGAGGAAGGAGGATTAGAGTGTGGTCCTTACGGTAAAGTTGGAATCGTTGGAGGAATGATTAC<br>TGGTGCAGAATCTACTCGCTTGGATTCAACTGATTCTGAGGGAAAAAAACATTTGTCATTAACAACTGGA<br>CTGCCATTTGGTGGTACATTAGCTGCGGGTATGACAATTGCACCAGGATTTAGAGCAGAGCTAGGTGTTA<br>TGTACCTTAGAAATATAAGCGCTGAGGTTGAAGTAGGTAAAGGCAAGGTAGATTCTAAAGGTGAGATAAA<br>GGCAGATTCTGGAGGTGGGACAGATACTCCTATACGTAAGCGGTTTAAACTTACACCACCTCAGCCTACT<br>ATAATGCCTATAAGTATAGCTGATCGTGATGTGGGGGTTGATACTGATATTCTTGCTCAAGCTGCTGCTG<br>GGCAACCACAGCTTACTGTTGAGCAGCGGGCTGCAGATAGGATTGCTTGGTTGAAGAATTATGCTGGTAT<br>TGACTATATGGTCCCAGATCCTCAGAATCCTAATGCTAGAGTTATAAATCCTGTATTGTTAAATATTACT<br>CAAGGGCCACCTAATGTACAGCCTAGACCTCGGCAAAATCTTGACATACTTGACCATGGTCAGTGGAGAC<br>ATTTGGTAGTTGGTGTTACTGCATTGTCACATGCTAATAAACCTAGCGTTACTCCTGTCAAAGTATTAAG<br>TGACAAAATTACTAAGATATATAGTGATATAAAGCCATTTGCTGATATAGCTGGTATTGATGTTCCTGAT<br>ACTGGTTTGCCTAATAGTGCATCTGTCGAACAGATACAGAGTAAAATGCAAGAATTAAACGATGTATTGG<br>AAGACCTCAGAGATTCTTTTGATGGGTATATGGGTAATGCTTTTGCTAATCAGATACAGTTGAATTTTGT<br>CATGCCGCAGCAAGCACAGCAGCAGCAGGGGCAAGGGCAGCAACAGCAAGCTCAAGCTACAGCGCAAGAA<br>GCAGTAGCAGCAGCAGCTGTTAGGCTTTTAAATGGCAATGATCAGATTGCGCAGTTATATAAAGATCTTG<br>TTAAATTGCAGCGTCATGCAGGAGTTAAGAAAGCCATGGAAAAATTAGCTGCCCAACAAGAAGAAGATGC<br>AAAGAATCAAGGTGAAGGTGACTGTAAGCAGCAACAAGGAGCATCTGAAAAATCTAAAGAAGGAAAAGGC<br>AAAGAAACAGAGTTTGATCTGAGTATGATTGTTGGCCAAGTTAAACTCTATGCTGACTTATTTACAACTG<br>AATCATTCTCAATATATGCTGGTGTTGGTGCAGGGTTAGCTCATACTTATGGAAAAATAGATGATAAGGA<br>TATTAAAGGGCATACAGGCATGGTTGCATCAGGAGCACTTGGTGTAGCAATTAATGCTGCTGAGGGTGTA<br>TATGTGGACTTAGAAGGTAGTTATATGCACTCATTCAGTAAAATAGAAGAGAAGTATTCAATAAATCCTC<br>TTATGGCAAGTGTAGG	1556 bp
RPA (1115F-1419R)	**GATCTTGTTAAATTGCAGCGTCATGCAGGA**GTTAAGAAAGCCATGGAAAAATTAGCTGCCCAACAAGAA<br>GAAGATGCAAAGAATCAAGGTGAAGGTGACTGTAAGCAGCAACAAGGAGCATCTGAAAAATCTAAAGAAG<br>GAAAAGGCAAAGAAACAGAG**TTTG**ATCTGAGTATGATTGTTGGCCAAGTTAAACTCTATGCTGACTTATTT<br>ACAACTGAATCATTCTCAATATATGCTGGTGTTGGTGCAGGGTTAGCTCATACTTATGGAAAAATAGATG<br>ATAAGGATATTAAAGGGCATACAGG**CATGGTTGCATCAGGAGCACTTGGTGTAGCA**	336 bp

[#]Sequence underline represent the nucleotide bases used to design CRISPR RNA (crRNA) for target detection. Sequence in purple colour is PAM sequence.
